# The EJC Binding and Dissociating Activity of PYM Is Regulated in *Drosophila*

**DOI:** 10.1371/journal.pgen.1004455

**Published:** 2014-06-26

**Authors:** Sanjay Ghosh, Ales Obrdlik, Virginie Marchand, Anne Ephrussi

**Affiliations:** European Molecular Biology Laboratory, Heidelberg, Germany; New York University, United States of America

## Abstract

In eukaryotes, RNA processing events in the nucleus influence the fate of transcripts in the cytoplasm. The multi-protein exon junction complex (EJC) associates with mRNAs concomitant with splicing in the nucleus and plays important roles in export, translation, surveillance and localization of mRNAs in the cytoplasm. In mammalian cells, the ribosome associated protein PYM (*Hs*PYM) binds the Y14-Mago heterodimer moiety of the EJC core, and disassembles EJCs, presumably during the pioneer round of translation. However, the significance of the association of the EJC with mRNAs in a physiological context has not been tested and the function of PYM *in vivo* remains unknown. Here we address PYM function in *Drosophila*, where the EJC core proteins are genetically required for *oskar* mRNA localization during oogenesis. We provide evidence that the EJC binds *oskar* mRNA *in vivo*. Using an *in vivo* transgenic approach, we show that elevated amounts of the *Drosophila* PYM (*Dm*PYM) N-terminus during oogenesis cause dissociation of EJCs from *oskar* RNA, resulting in its mislocalization and consequent female sterility. We find that, in contrast to *Hs*PYM, *Dm*PYM does not interact with the small ribosomal subunit and dismantles EJCs in a translation-independent manner upon over-expression. Biochemical analysis shows that formation of the PYM-Y14-Mago ternary complex is modulated by the PYM C-terminus revealing that *Dm*PYM function is regulated *in vivo*. Furthermore, we find that whereas under normal conditions *Dm*PYM is dispensable, its loss of function is lethal to flies with reduced *y14* or *mago* gene dosage. Our analysis demonstrates that the amount of *Dm*PYM relative to the EJC proteins is critical for viability and fertility. This, together with the fact that the EJC-disassembly activity of *Dm*PYM is regulated, implicates PYM as an effector of EJC homeostasis *in vivo*.

## Author Summary

The multi-protein exon junction complex (EJC) is deposited at exon-exon junctions on mRNAs upon splicing. EJCs, with Y14, Mago, eIF4AIII and Barentsz proteins at their core, are landmarks of the nuclear history of RNAs and play important roles in their post-transcriptional regulation. In mammalian cells, the Y14-Mago interacting protein PYM associates with ribosomes and disassembles EJCs in the cytoplasm. However, the physiological function of PYM and its regulation *in vivo* remains unknown. We have analysed PYM function during *Drosophila* oogenesis, where the EJC is essential for *oskar* mRNA localization in the oocyte, a prerequisite for embryonic patterning and germline formation. We find that *Drosophila* PYM interacts with Y14-Mago but, in contrast to mammalian PYM, does not bind ribosomes. We demonstrate that EJCs associated with *oskar* mRNA *in vivo* are disassembled by PYM over-expression in a translation-independent manner, causing *oskar* mislocalization. Our *in vivo* analysis shows that the *Drosophila* PYM C-terminal domain modulates PYM-Y14-Mago interaction, revealing that PYM is regulated in *Drosophila*. Furthermore, PYM is essential for viability of flies lacking one functional copy of *y14* or *mago*, supporting a role of PYM in EJC homeostasis. Our results highlight a distinct mode of regulation of the EJC-dissociating protein PYM in *Drosophila*.

## Introduction

In eukaryotes, post-transcriptional regulation of gene expression plays important roles in development and differentiation. These include RNA processing events in the nucleus, such as splicing, which also affects 3′ end processing of the RNA, mRNA export, localization, translational enhancement and decay [1]–[5]. The multi-protein exon junction complex (EJC), which is recruited to RNAs upon splicing, has been linked to most of these steps in RNA maturation. The EJC assembles 20–24 nucleotides (nt) upstream of splice junctions and is organized around a core complex of four proteins: the DEAD box RNA helicase eIF4AIII, which is deposited by the spliceosomal protein CWC22 [6]–[8] and binds the mRNA independently of its sequence, the Y14 (Tsunagi)-MAGOH (Mago nashi, Mago) heterodimer, which stabilizes the complex, and MLN51 (Barentsz, Btz), which associates with the EJC upon RNA export [9].

In *Drosophila*, asymmetric localization of several key mRNAs during oogenesis is essential for embryonic patterning [10]. While in transport, these mRNAs are translationally repressed, and protein is produced only upon mRNA localization and at a particular developmental stage. The localization of *oskar* mRNA to the posterior pole of the oocyte requires splicing and the EJC core proteins [4], [11]–[15], indicating that nuclear events determine mRNA targeting within the cytoplasm. However, *in vivo* association of an assembled EJC with *oskar* has not been shown and the basis for the requirement of the complex in RNA transport remains unclear.

Partner of Y14-Mago (PYM) was identified through its association with the Y14-Mago heterodimer in *Drosophila* S2 cells [16]. The crystal structure of the PYM-Y14-Mago trimeric complex revealed that the PYM N-terminal residues are necessary for its interaction with Y14-Mago [17]; in mammals, this interaction can provoke disassembly of the EJCs from spliced mRNAs [18]. Furthermore, in HeLa cells, the PYM C-terminus, which bears similarity to eIF2A, associates with the 40S ribosomal subunit in the cytoplasm [19]. These observations led to the proposal that cytosolic ‘free’ PYM binds ribosomes and dislodges EJCs from mRNAs during the pioneer round of translation, thus restricting EJC disassembly to translating mRNAs. However, the function of PYM and its relationship to the EJC has not been characterized *in vivo*.

In this study, we characterize the function of PYM during *Drosophila* oogenesis. We show that *Drosophila* PYM (*Dm*PYM) binds Y14-Mago but that, in contrast to its mammalian ortholog, it does not appear to interact with ribosomes. While *Dm*PYM is not required for viability, it is essential in flies lacking one functional copy of *y14* or *mago*. We demonstrate that over-expression of the N-terminus of *Dm*PYM in the ovary is sufficient to dissociate EJCs from mRNAs in the cytoplasm independently of translation and causes female sterility due to *oskar* mislocalization in the oocyte. Finally, we show that assembly of the PYM-Y14-Mago ternary complex is modulated by the PYM C-terminal domain, indicating that PYM activity is controlled by a distinct mechanism in *Drosophila*.

## Results

### PYM is a non-essential gene in *Drosophila*

**Figure 1:**
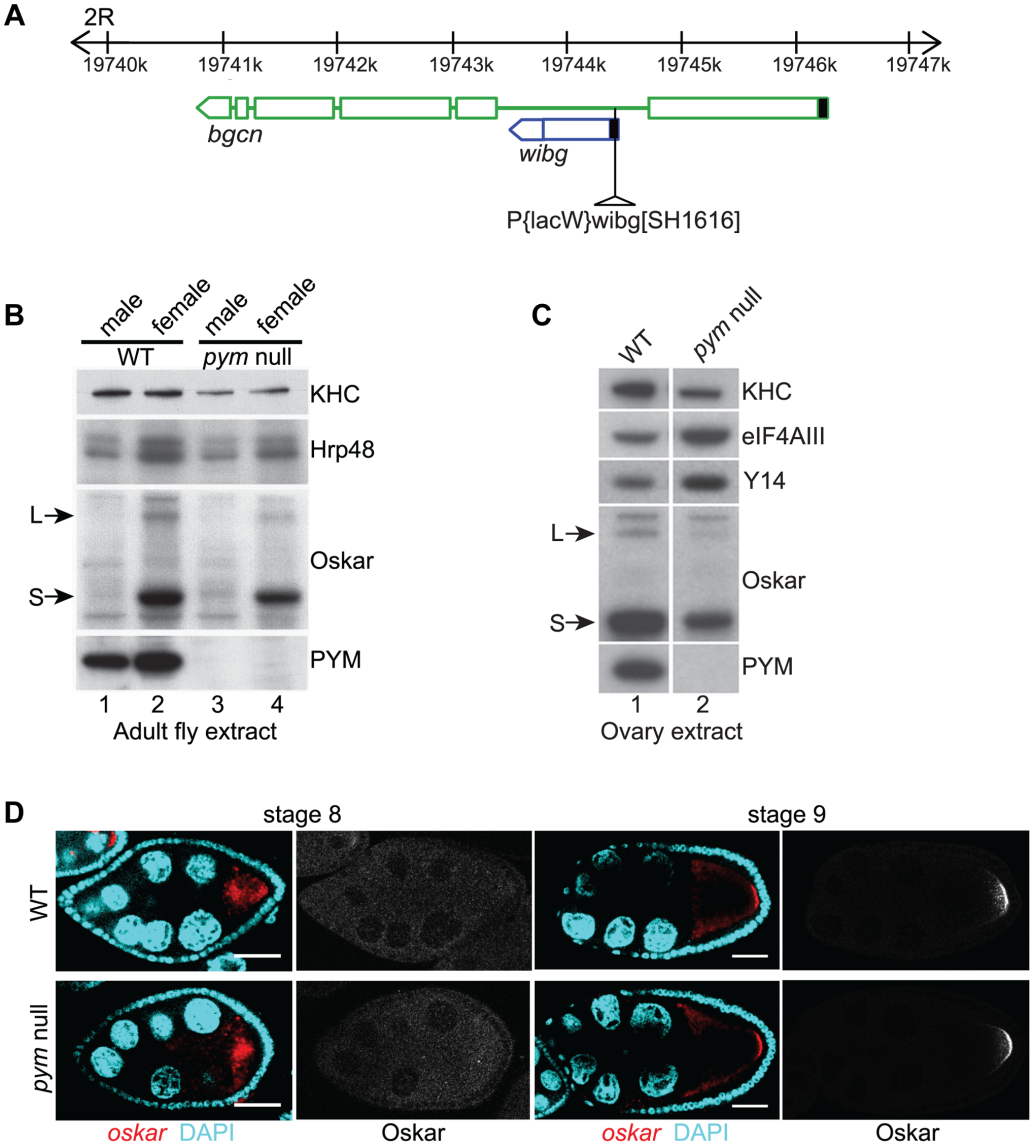
PYM is a non-essential gene in *Drosophila*. (A) Schematic diagram showing the genomic organization of *pym* (*wibg*, shown in blue) relative to the *bgcn* gene (show in green) in the right arm of the second chromosome (2R). The centromere is to the left and the telomere is at the right. Open boxes and interconnecting lines represent exons and introns, respectively. The 5′UTRs are shown as filled black boxes. The insertion site of the P-element, *P{lacW}wibg^SH1616^* is depicted as a triangle. (B and C) Western blot analysis of *Drosophila* adult (B) and ovary (C) extracts shows the absence of PYM protein from *pym* null flies (B, lanes 3 and 4; C, lane 2) as compared to the wild-type (WT; B, lanes 1 and 2; C, lane 1). The antibodies used for staining are indicated on the right of the panel. S = short Oskar, L = long Oskar, KHC = kinesin heavy chain. (D) Fluorescent *in situ* hybridization coupled with immunostaining of wild-type (WT, upper panel) and *pym* null (lower panel) egg-chambers during stages 8 and 9 of oogenesis. *oskar* mRNA is detected with a 3′UTR probe (red) while anti-Oskar staining is shown in greyscale. DAPI is in cyan. Scale bar 25 µm.

*Drosophila pym* (*wibg*, CG30176), situated within intron 1 of the *bgcn* gene (Figure 1A) [20], is expressed in the ovary, and the protein maternally deposited in the embryo (Figure 1C, lane 1 and Figure S1A, lane 1) [21]. Immunostaining of ovaries revealed that *Dm*PYM is present in the germarium, nurse cell and follicle cell cytoplasm, and within the oocyte is uniformly distributed in the cytoplasm (Figure S1B). A cytoplasmic distribution of PYM has also been reported in *Drosophila* S2, HeLa and plant cells [17], [19], [22].

To assess the role of PYM *in vivo*, we made use of a *Drosophila* line bearing a P element insertion in the gene (*P{lacW}wibg^SH1616^*) that constitutes a molecular null allele of *pym* (Figure 1B; Figure S1E). The flies were viable, although the females displayed defective ovarian development, due to impaired *bgcn* function. Transgenic expression of a *bgcn* cDNA tagged with GFP (*bgcn*GFP) restored normal oogenesis, although no PYM protein nor *pym* RNA was detected (Figure 1C, lane 2; Figure S1A, lane 2; Figure S1E). We used such flies, to which we refer as “*pym* null”, for our subsequent analyses (Table S1). Severe knockdown of PYM in ovaries and early embryos (Figure S1A, lanes 3 and 4) by expression of shRNAs targeting *pym* in the female germline [23] also did not appear to affect oogenesis or embryonic development.

In HeLa cells, PYM (*Hs*PYM) enhances translation of intron-containing reporter mRNAs and is required to stimulate translation of intronless herpesvirus gM transcript by ORF57 protein during the lytic cycle [19], [24]. In addition, *Hs*PYM knockdown resulted in increased association of EJC with spliced reporter mRNAs [18], implying a role of PYM in EJC removal. As *oskar* mRNA transport to the oocyte posterior pole requires the EJC core proteins, and tight control of *oskar* translation is critical for normal embryonic development [11]–[15], [25], we examined the distribution of *oskar* mRNA and protein in *pym* null egg-chambers. As shown in Figure 1D, *oskar* mRNA was transported into the oocyte during the early stages of oogenesis and accumulated at the posterior pole in of the oocyte at stages 8–9 and Oskar protein was first detected at the posterior pole at stage 9, when the mRNA is localized. Localization of *gurken* and *bicoid* mRNAs, as well as expression of Gurken protein, also appeared normal in *pym* null egg-chambers (Figure S1C, D).

In *Drosophila* S2 cells and HeLa cells, PYM interacts with the Y14-Mago heterodimer [17]–[19] which, together with eIF4AIII and Btz, constitute the EJC core [26], [27]; in *Arabidopsis thaliana*, PYM (*At*PYM) interacts both with the heterodimer and with Y14 and Mago monomers [22]. Furthermore, in mammalian cells, PYM over-expression has been shown to destabilize EJCs [18]. To probe whether *Dm*PYM might have a role in EJC regulation *in vivo*, we performed genetic interaction tests between *pym* and EJC components in the fly (Table S1). *y14*, *mago* or *eIF4AIII* heterozygous mutant flies, like *pym* null flies, are viable and fertile. Remarkably however, we failed to generate *pym* null flies harbouring only one functional copy of *y14* or *mago*. In contrast, *pym* null, *eIF4AIII* heterozygous flies were viable. The lethality we observed was specific, as transgenic expression of FLAG-tagged Y14 at close to endogenous levels rescued the lethality of the *pym* null, *y14* heterozygous flies. These results show that *Dm*PYM function is essential when Y14 and Mago are present at reduced levels, indicating an important relationship between PYM and these EJC components *in vivo*.

### *Drosophila* PYM interacts with Y14-Mago but not with ribosomes

**Figure 2:**
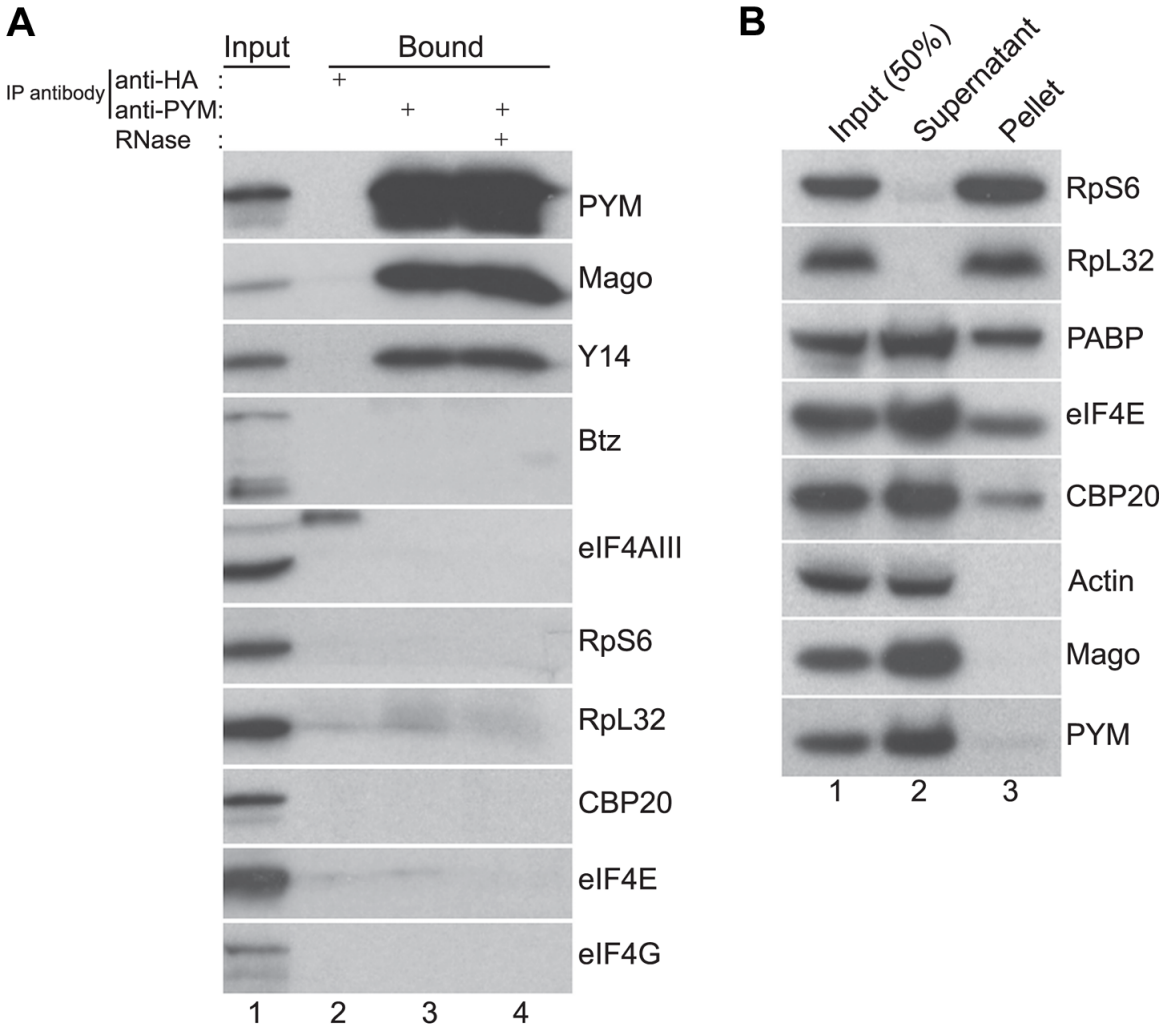
Endogenous *Dm*PYM interacts with Y14 and Mago but not with ribosomes. (A) Immunoprecipitation using anti-HA (lane 2) and anti-PYM (lane 3) antibody was performed using wild-type ovarian extracts. The precipitated proteins were analyzed by western blotting and stained with the antibodies indicated at the right of the panel. Lane 4 shows the anti-PYM precipitate from an extract treated with RNase. Input (1%) is shown in lane 1. (B) Sucrose cushion centrifugation of wild-type cytoplasmic ovarian extract. The input (lane 1; 50%), supernatant (lane 2) and pellet (lane 3) fractions were processed for western blot analysis and stained with the antibodies indicated at the right of the panel.

To determine the endogenous binding partners of *Dm*PYM during oogenesis, we performed co-immunoprecipitations (coIPs) from cytoplasmic extracts of wild-type ovaries. As shown in Figure 2A, the EJC core proteins Y14 and Mago co-precipitated with PYM when using an anti-PYM antibody, but not an unrelated antibody, demonstrating specificity of the interaction. In contrast, eIF4AIII and Btz did not co-precipitate. Addition of RNase during the coIP did not affect Y14 and Mago recovery, indicating that *Dm*PYM binds to Y14 and Mago by direct protein-protein interaction. This is consistent with a previous study showing that a 35 residue N-terminal domain of *Drosophila* PYM interacts with Y14 and Mago at their heterodimerization interface [17]. Indeed, the amino acid residues of PYM necessary for this interaction are conserved across metazoa (Figure S2A).

In HeLa cells, over-expressed PYM interacts with the 48S pre-initiation complex, and components of the eIF4F complex, ribosomal proteins, and translation factors such as CBP80 and PABP coIP with *Hs*PYM [19]. Furthermore, sucrose density gradient analysis revealed that the *Hs*PYM C-terminus, which shows a high degree of homology to *Hs*eIF2A, is necessary for co-sedimentation with ribosomal fractions [18], [19]. Thus it was proposed that PYM physically links the EJC to the translation machinery, enhancing translation of spliced mRNAs [18], [19]. To test whether the endogenous *Drosophila* PYM associates with ribosomal subunits, we performed sucrose cushion centrifugation to pellet the ribosomes from ovarian cytoplasmic extracts and examined the distribution of the endogenous PYM by western blot analysis. As shown in Figure 2B, ribosomal proteins were enriched in the pellet, whereas actin, a predominantly cytoplasmic protein, fractionated in the post-ribosomal supernatant, validating the assay. Cap-binding proteins and PABP were also detected in the pellet, indicating the presence of translationally competent mRNPs in this fraction. In contrast, the quasi-totality of *Dm*PYM was recovered in the post-ribosomal supernatant (Figure 2B), suggesting a lack of interaction with ribosomes. In addition, coIP assays using an anti-PYM antibody failed to reveal a significant interaction of *Dm*PYM with ribosomal subunits or components of the translation initiation complex, as compared with Y14 and Mago (Figure 2A). This suggests that, unlike *Hs*PYM, *Dm*PYM does not associate with the translation initiation machinery. In addition, the absence of a significant association with CBP20 and eIF4E excludes a major role of PYM in cap-dependent translation regulation in *Drosophila*.

### *Dm*PYM over-expression disrupts *oskar* localization

To analyze the function of the *Dm*PYM during *Drosophila* oogenesis, we divided the protein into N-terminal (N), middle (M), and C-terminal (C) domains, and generated a set of eGFP-tagged PYM deletion transgenes (Figure S3A). Upon expression in the female germline, the bulk of the GFP signal in the PYM-GFP egg-chambers was distributed uniformly throughout the cytoplasm of the nurse cells (Figure S3B), similar to endogenous PYM (Figure S1B); however, in the case of N-, M- and C-PYM, some GFP signal was also detected in the nurse cell nuclei.

In spite of their similar distribution, the different PYM-GFP proteins had dramatically different effects on embryonic development. Females expressing FL-PYM or ΔN-PYM in the germline were fertile (Table S1). In contrast, those expressing ΔC- and N-PYM had reduced fertility: most of the progeny embryos failed to hatch due to abdominal patterning defects, and those that did hatch developed into sterile adults. Such a “grandchildless” phenotype is suggestive of reduced Oskar protein function. Indeed, immunoblot analysis of ovaries of PYM transgenic females confirmed that Oskar protein levels were substantially reduced in ΔC- and N-PYM-GFP expressing ovaries, compared with FL- or ΔN-PYM-GFP expressing, or wild-type ovaries (Figure S4A). This suggested that expression of the PYM N-terminus interferes with Oskar expression.

**Figure 3:**
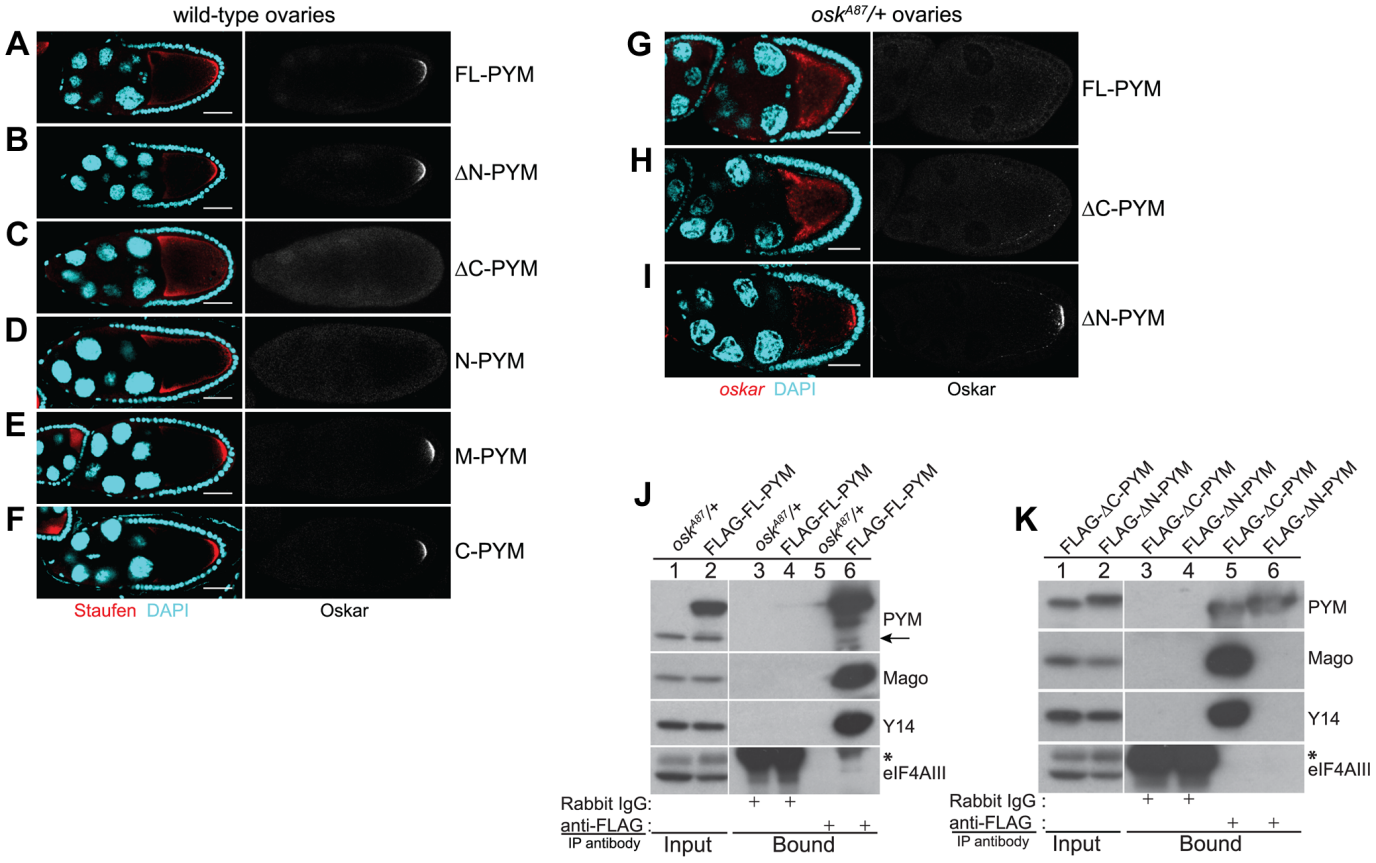
Over-expression of the N-terminal of *Dm*PYM affects *oskar* transport. (A–F) Distribution of Staufen (red, left panel) and Oskar (greyscale, right panel) proteins as revealed by immunostaining of wild-type stage 9 egg-chambers expressing GFP-tagged PYM transgenes as indicated to the right of the panel. DAPI is in cyan. Scale bar 25 µm. (G–I) Fluorescent *in situ* hybridization and immunostaining showing the distribution pattern of *oskar* mRNA (red; left panel) and Oskar protein (greyscale; right panel) in *osk^A87^*/+ egg-chambers expressing FLAG-FL-PYM (G), FLAG-ΔC-PYM (H), or FLAG-ΔN-PYM (I). *oskar* mRNA was detected using a *oskar* 3′UTR probe. DAPI is shown in cyan. Scale bar 25 µm. (J and K) Immunoprecipitation from cytoplasmic extracts from *osk^A87^/+* ovaries expressing FLAG-tagged PYM proteins using mouse anti-FLAG antibody. The protein precipitates from *osk^A87^/+* (J, lanes 3 and 5), and *osk^A87^/+* expressing FL-PYM (J, lanes 4 and 6), ΔN-PYM (K, lanes 4 and 6), or ΔC-PYM (K, lanes 3 and 5) ovarian extracts were western blotted and probed with the antibodies indicated at the right of the panels. The inputs (1%) are shown in lanes 1 and 2 of the panels. Endogenous PYM is indicated by an arrow. The electrophoretic mobility of the FLAG-ΔN- and ΔC-PYM proteins in K is indistinguishable from that of the endogenous PYM protein. An asterisk denotes the IgG heavy chain.

Expression of the posterior determinant *oskar* is spatio-temporally controlled such that Oskar protein is produced and accumulates stably only upon localization of the mRNA at the posterior pole of the oocyte during mid-oogenesis [28]–[30]. The low levels of Oskar protein in ΔC- and N-PYM expressing ovaries could therefore be due to a failure in *oskar* mRNA localization or translation at the posterior pole. To distinguish between these possibilities, we examined the distribution of the *oskar* mRNP component Staufen and of Oskar protein by immunostaining. As shown in Figure 3C, D, Staufen failed to enrich at the posterior pole of ΔC- and N-PYM oocytes indicating a failure in *oskar* mRNA localization (see also Table S1), and Oskar protein was not detected in these oocytes during oogenesis, consistent with the western blot analysis (Figure S4A). These results show that over-expression of the *Dm*PYM N-terminal domain is sufficient to disrupt posterior localization of *oskar* and thus explains the absence of Oskar protein and the consequent female sterile phenotype of ΔC- and N-PYM expressing females.

We also noted that *oskar* localization was somewhat impaired in FL-PYM expressing egg-chambers: although Staufen accumulated at the oocyte posterior pole, the protein was also detected around the cortex (Figure 3, compare panel A with B, E and F), suggesting that, while less potent than ΔC- and N-PYM, FL-PYM also has some capacity to interfere with *oskar* transport.

To test if the amount of PYM relative to *oskar* mRNA and EJCs might be important for transport, we expressed FLAG-tagged FL-, ΔN- and ΔC-PYM transgenes in the germline of *osk^A87^*/+ females, which produce only half the normal dose of *oskar* mRNA [31] (Table S1). Both FL-PYM and ΔC-PYM transgenes caused *oskar* mislocalization, and no Oskar protein was detected in the *osk^A87^*/+ oocytes (Figure 3G, H). Western blot analysis revealed a substantial reduction in Oskar protein levels in FL- and ΔC-PYM expressing ovaries, as compared with ΔN-PYM ovaries or the wild-type control (Figure S4B, C). Both FL- and ΔC-PYM expressing females produced embryos with a strong posterior group phenotype (data not shown); only ∼5% of embryos produced by FL-PYM females hatched, and these developed into sterile adults.

In contrast to *oskar*, *gurken* and *bicoid* mRNAs were correctly localized to the antero-dorsal corner and anterior cortex of the PYM over-expressing oocytes (Figure S4D). The mislocalization of *oskar* mRNA upon PYM over-expression was independent of the tag and its position in the protein, as expression of PYM transgenes tagged at their C-terminus with eGFP produced a similar effect (data not shown). All subsequent analyses of PYM function in the flies were performed using egg-chambers expressing epitope-tagged PYM constructs in the *osk^A87^/+* genetic background.

The presence of endogenous PYM does not appear to affect *oskar* localization in wild-type or *osk^A87^/+* egg-chambers. To assess if the observed effects of PYM over-expression on *oskar* localization might be due to aberrant interaction of the over-expressed PYM proteins with EJC components, we performed anti-FLAG coIPs from cytoplasmic extracts from ovaries expressing the different FLAG-PYM transgenes. As observed for endogenous PYM (Figure 2A), FL- and ΔC-PYM proteins co-precipitated Y14 and Mago, but not eIF4AIII, whereas ΔN-PYM did not interact with any of the EJC core proteins tested (Figure 3J, K). These data, consistent with previous observation in HeLa cells [18], [19], show that the N-terminal portion of *Dm*PYM exclusively mediates its interaction with the Y14-Mago heterodimer.

To test for possible interaction of over-expressed PYM proteins with the ribosomal subunits, we performed coIPs from cytoplasmic extract of the GFP-tagged PYM expressing ovaries. As shown in Figure S5A, similar to the endogenous protein, the over-expressed PYM proteins did not show significant interaction with either of the ribosomal subunits. Consistent with this, sucrose cushion centrifugation assays revealed that the tagged PYM proteins predominantly fractionated in the post-ribosomal supernatant (Figure S5B). Importantly, the GFP-tagged FL-PYM detected in the ribosomal pellet fraction was not associated with the small ribosomal subunit (Figure S5B, lane 10), as treatment of the extract with EDTA, which led to redistribution of the small ribosomal subunit to the supernatant fraction, did not affect FL-PYM distribution (Figure S5C, lane 10). We conclude that epitope-tagging does not affect the interactions of PYM or its distribution *in vivo*.

Taken together, our data show that increased levels of *Dm*PYM relative to *oskar* mRNPs disrupt localization of the mRNA. Furthermore, this property of *Dm*PYM is mediated by its N-terminal domain and is down-regulated by the C-terminal domain.

### Increasing PYM dosage causes EJC dissociation from *oskar* mRNA

**Figure 4:**
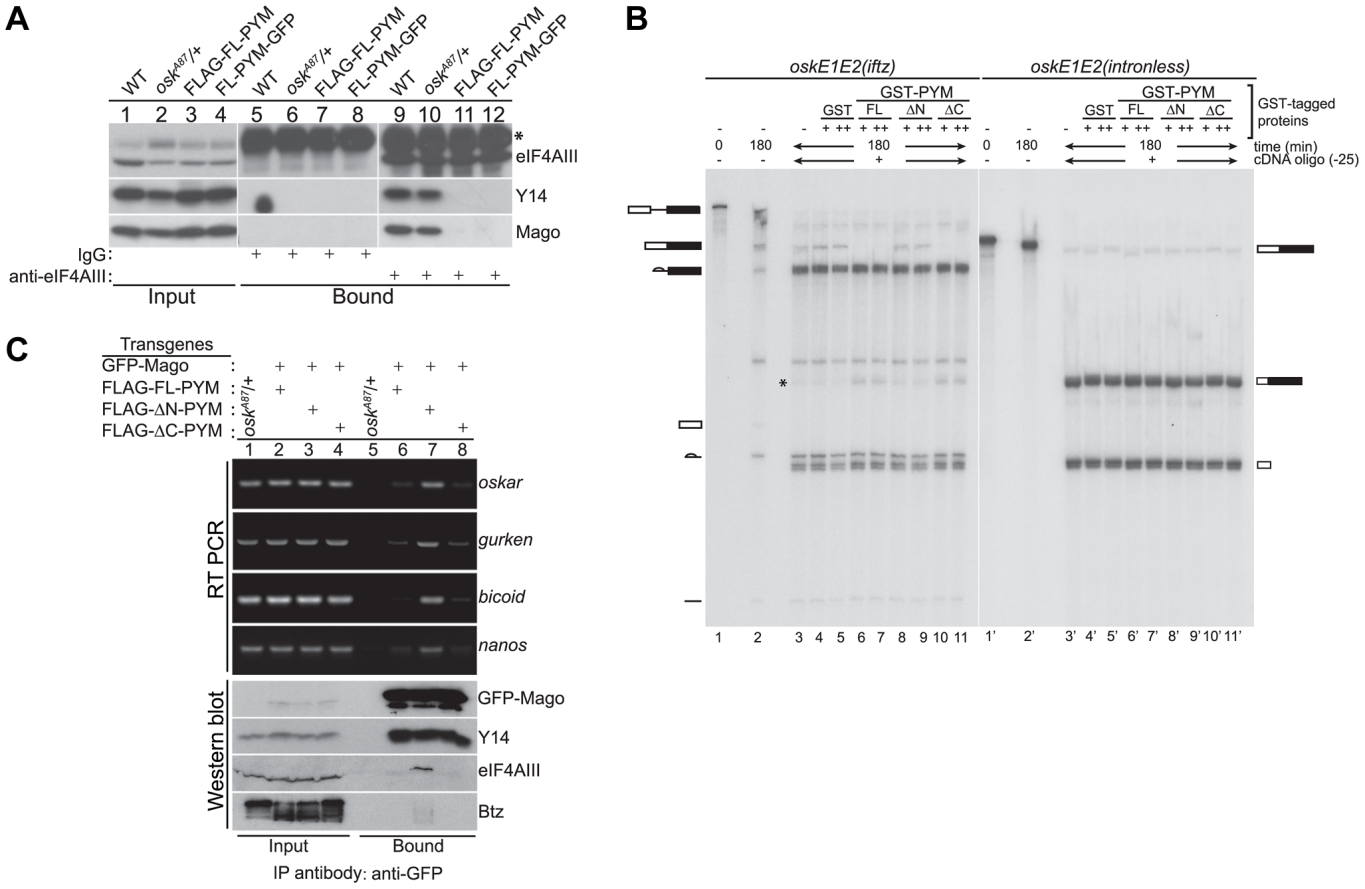
Ectopic *Dm*PYM disassembles EJC on the mRNAs. (A) Cytoplasmic ovarian extract from wild-type (WT), *osk^A87^/+*, and *osk^A87^/+* flies expressing FLAG- or GFP-tagged FL-PYM were immunoprecipitated using anti-eIF4AIII antibody (lanes 9–12) or rabbit IgG (lanes 5–8). The inputs (1%, lanes 1–4) and the bound protein samples were analyzed by western blotting using antibodies indicated at the right of the panel. An asterisk indicates the heavy chain of IgG. (B) *In vitro* splicing of ^32^P-labelled *oskE1E2(iftz)*(lanes 2–11) and *oskE1E2(intronless)* (lanes 2′–11′) RNAs was carried out using embryo nuclear extract for 180 min. Aliquots of the reactions were supplemented with buffer (lanes 3 and 3′), GST (0.5 µM (+, lanes 4 and 4′) and 1 µM (++, lanes 5 and 5′)), GST-FL-PYM (0.5 µM (+, lanes 6 and 6′) and 1 µM (++, lanes 7 and 7′)), GST-ΔN-PYM (0.5 µM (+, lanes 8 and 8′) and 1 µM (++, lanes 9 and 9′)) or GST-ΔC-PYM (0.5 µM (+, lanes 10 and 10′) and 1 µM (++, lanes 11 and 11′)) and incubated for 30 min. An oligonucleotide centered at −25 relative to the first splice junction of *oskar* was added to elicit RNase H cleavage (lanes 3–11, 3′–11′) and the samples were resolved by urea PAGE. The presence of RNA cleavage products (indicated by an asterisk) in lanes 6, 7, 10 and 11 suggests loss of protection from RNase H cleavage due to disassembly of the EJC. The positions of the pre-mRNA, mRNA and splicing intermediates and products are shown at the sides of the panel. (C) Top panel: Semi-quantitative RT-PCR analysis of the mRNAs (indicated on the right of the panel) obtained by immunoprecipitation using GFP-Trap beads either from *osk^A87^/+* ovarian extracts or *osk^A87^/+* ovaries co-expressing GFP-tagged Mago and one of the FLAG-tagged PYM constructs, as indicated at the top of the panel. Lanes 1–4 show the input samples, and lanes 5–8 show mRNAs recovered in the immunoprecipitates. Bottom panel: Western blot of the samples used for RT-PCR analysis stained with antibodies indicated at the right of the panel. The GFP-Mago panel was probed with anti-GFP antibody. 20% of the input and bound fractions from the immunoprecipitate was used for western analysis.

Although the *in vivo* association of the EJC with *oskar* mRNA has never been demonstrated, the EJC core proteins are considered to be essential components of *oskar* mRNPs [4], [11], [14]. We therefore hypothesized that *oskar* mislocalization upon PYM over-expression was due to the loss of EJC association with the mRNA, and assessed the overall integrity of EJCs in the ovary. Over-expression of either FLAG-FL-PYM or FL-PYM-GFP in *osk^A87^*/+ ovaries caused a substantial reduction in coIP of Y14 and Mago with eIF4AIII (Figure 4A, compare lanes 9 and 10 with lanes 11 and 12), indicating disassembly of the EJC core complex. The interaction of eIF4AIII with Mago was also impaired in egg-chambers over-expressing ΔC-PYM, but not ΔN-PYM (Figure 4C, lower panel, lanes 7 and 8). Hence, over-expression of the *DmPYM* N-terminal domain causes disassembly of the *Drosophila* EJC core. The fact that in the absence of PYM over-expression intact EJCs are recovered (Figure 4A, lanes 9 and 10) confirms that endogenous levels of *Dm*PYM are not deleterious to EJC integrity.

To test whether elevated levels of FL- or ΔC-PYM cause the EJC to dissociate from *oskar* mRNA, we first performed *in vitro* splicing assays coupled with RNase H cleavage using *osk(E1E2(iftz))* RNA and assessed the degree of protection of the EJC binding site, in the presence or absence of recombinant PYM proteins [32]. Briefly, after the completion of splicing and concomitant EJC deposition, reactions were incubated with GST-tagged FL-, ΔN- or ΔC-PYM, followed by RNase H cleavage induced by an oligonucleotide (Ol-25) complementary to the EJC deposition site. As shown in Figure 4B, a significant decrease of *oskar* mRNA correlated with an increase of the corresponding RNase H cleavage product was observed in the presence of GST-FL- and ΔC-PYM (lanes 6, 7 and 10, 11, respectively; * shows the position of the cleavage product), compared to GST-ΔN-PYM or the GST control, indicating a loss of EJC binding. This result shows that elevated amounts of *Dm*PYM can cause mature EJCs to dissociate from *oskar* mRNA *in vitro*, raising the possibility that *oskar*-associated EJCs are destabilized by ectopic PYM *in vivo*.

To test directly whether high PYM levels cause the EJC to dissociate from *oskar* mRNA *in vivo*, we co-expressed GFP-Mago and FLAG-tagged PYM constructs in the germline of *osk^A87^/+* flies and performed RNA-coimmunoprecipitation (RIP) from cytoplasmic extracts of ovaries. The EJC was immunoprecipitated using GFP-Trap beads and the precipitated RNA extracted for semi-quantitative RT-PCR analysis. As shown in Figure 4C, *oskar* mRNA was enriched in immunoprecipitates of ΔN-PYM extract, demonstrating that EJCs are assembled on *oskar* mRNA *in vivo* and that over-expression of the C-terminal and middle region of *Dm*PYM does not alter its integrity. In contrast, although similar amounts of GFP-Mago were recovered, considerably less *oskar* mRNA was immunoprecipitated from FL-PYM or ΔC-PYM ovaries (Figure 4C). These results show that over-expression of the *Dm*PYM N-terminal domain causes EJC dissociation from *oskar* mRNA in the ovary. We further investigated whether the association of the EJC with other localized mRNAs was also affected by the expression of PYM transgenes. Interestingly, a profile similar to *oskar* was observed with *bicoid*, *nanos* and *gurken* mRNAs: these mRNAs were also co-precipitated with a considerably lower efficiency from FL-PYM and ΔC-PYM, compared with ΔN-PYM extracts, demonstrating that the N-terminus of PYM is sufficient to dissociate the EJC from the spliced mRNAs *in vivo*. Since FL-PYM or ΔC-PYM over-expression leads to *oskar* mislocalization in the oocyte (Figure 3G, H), but not that of *bicoid* or *gurken* (Figure S4D), we conclude that although the EJC associates with *bicoid* and *gurken* mRNAs in the oocyte, its function is dispensable for their localization. This is consistent with normal localization of *bicoid* and *gurken* in *y14* mutant oocytes [11].

### Ectopic PYM dissociates EJCs independent of the elongating ribosomes

**Figure 5:**
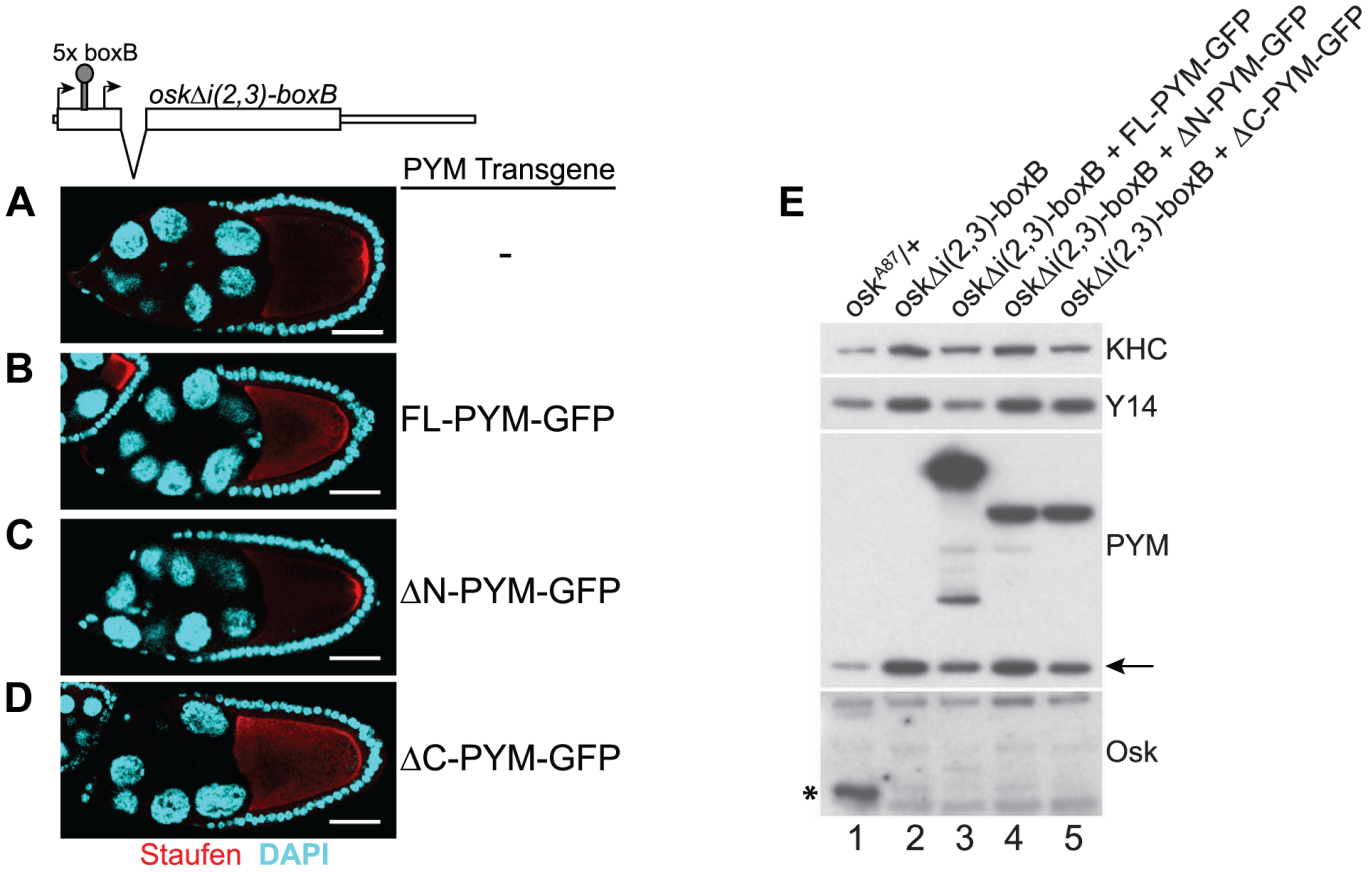
PYM disassembles EJC from a non-translatable *oskar* mRNA. (A–D) Whole mount immunostaining using anti-Staufen antibody (red) of stage 9 *oskar* RNA null egg-chambers expressing either *oskΔi(2,3)-boxB* transgene alone (A) or together with the GFP-tagged PYM transgenes (B–D) indicated at the right of the panels. A schematic diagram of the *oskΔi(2,3)-boxB* RNA is shown at the top of the panel. DAPI is in cyan. Scale bar 25 µm. (E) Western blot analysis of ovarian extract of flies shown in A–D shows the absence of Oskar translation in egg-chambers expressing *oskΔi(2,3)-boxB* transgene. Antibodies used for protein detection are indicated on the right. An asterisk indicates the short isoform of Oskar protein and the arrow shows endogenous PYM. KHC = kinesin heavy chain.

Previous studies have shown that *Hs*PYM interacts with the translation pre-initiation complex and disassembles mature cytoplasmic EJCs from spliced RNAs [18], [19]. Having found no evidence of association of *DmPYM* with ribosomal subunits (Figure 2 and Figure S5), yet shown that *Dm*PYM has the capacity to dismantle EJCs from mRNAs in the egg-chamber (Figure 4), we assessed whether this effect is translation-dependent. We monitored the distribution and translational output of *oskΔi(2,3)-boxB* transgenic mRNA [32] in PYM-GFP expressing egg-chambers. Although *oskΔi(2,3)-boxB* mRNA is spliced and localized (Figure 5A), it is not translated due to the presence of 5× boxB stem-loops between the two *oskar* start codons (Figure 5E, lane 2). As shown in Figure 5B–D, upon expression of FL- or ΔC-PYM, but not ΔN-PYM, *oskΔi(2,3)-boxB* mRNA was mislocalized in the oocyte, as revealed by anti-Staufen immunostaining. This demonstrates that increased levels of *Dm*PYM cause EJC dissociation from *oskar* mRNA in the absence of translation. Furthermore, these data provide evidence that the EJC is deposited on the *oskΔi(2,3)-boxB* mRNA in the oocyte and that integrity of the complex is essential for *oskar* transport.

### The C-terminus of *Dm*PYM modulates its interaction with Y14-Mago

**Figure 6:**
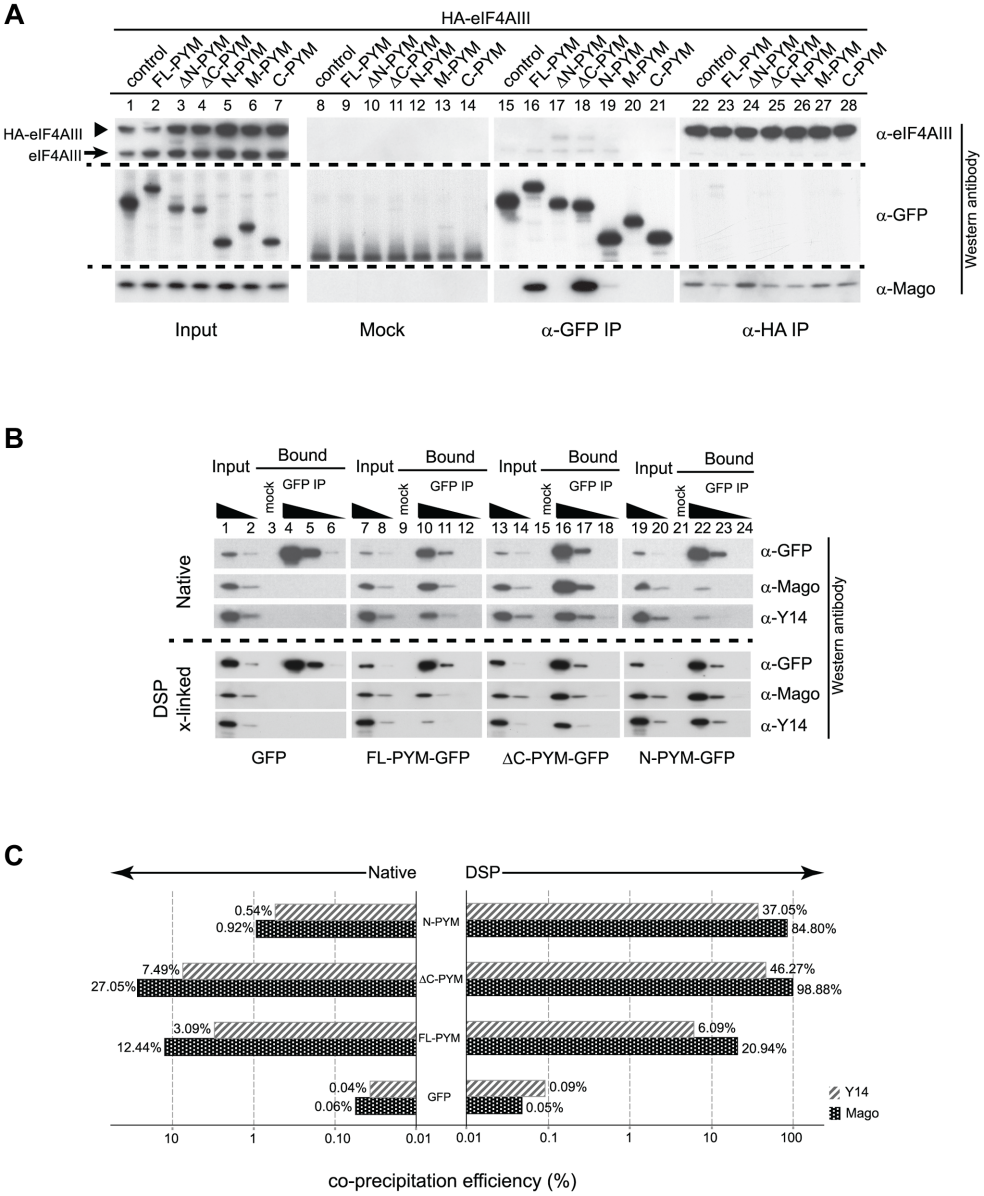
Regulation of PYM function by its domains. (A) Lysates of S2 cells co-expressing HA-eIF4AIII and GFP (control) or GFP-tagged PYM proteins (as indicated at the top of the panel) were immunoprecipitated using protein G (Mock), GFP-Trap (α-GFP IP) and anti-HA (α -HA IP) beads. Input panel shows 1.6% of the extracts and the bound fractions are shown in separate panels. The antibodies used for western analysis are indicated on the right of the panel. The anti-PYM antibody was not used for detection of PYM-GFP proteins due its preferential detection of the *Dm*PYM C-terminus (data not shown). Arrow and arrowhead indicate endogenous and HA-tagged eIF4AIII proteins, respectively. (B) Lysates of S2 cells expressing GFP (control) or GFP-tagged FL-, ΔC-, or N-PYM proteins were subjected to immunoprecipitation using protein G (Mock) or GFP-Trap (GFP IP) beads under native and DSP cross-linked conditions. 1.6% and 0.32% of inputs utilised in IPs were loaded in lanes 1, 7, 13, 19 and lanes 2, 8, 14, 20, respectively. Bound fractions (20%) were loaded in lanes 4, 10, 16, 22, and the corresponding 5× and 20× dilutions in lanes 5, 11, 17, 23 and lanes 6, 12, 18, 24 respectively. Lanes 3, 9, 15 and 21 contain 20% of the mock IP precipitates. Antibodies utilized for the western blot analysis are indicated at the right of the panels. (C) CoIP efficiencies of Mago and Y14 with GFP (control) or GFP-tagged FL-, ΔC- and N-PYM proteins under native (left panel) and DSP cross-linked (right panel) conditions. Mago and Y14 coIP efficiency is defined as a percentage of measured GFP enrichment in the corresponding GFP IPs. Plotted bar values represent the mean of two biological and four technical replicates.

To investigate the contribution of the different *Dm*PYM domains to EJC binding and disassembly, we performed coIPs from extracts of *Drosophila* S2 cells co-expressing HA-tagged eIF4AIII and either GFP (control) or GFP-tagged PYM proteins. To monitor both the PYM-Mago interaction and EJC integrity, PYM-GFP and HA-eIF4AIII were immunoprecipitated separately using GFP-Trap and HA-beads, respectively, and western blots of the bound fractions were probed with anti-Mago antibodies. Neither of the ectopically expressed proteins bound detectably to the agarose beads (Figure 6A, lanes 8–14, mock), demonstrating specificity of the assay.

Consistent with our previous experiments (Figures 3J, K and 4A, C), Mago co-precipitated exclusively with the FL-, ΔC- and N-PYM-GFP fusion proteins, which contain the N-terminal Y14-Mago binding domain, but not with ΔN-, M and C-PYM, or the GFP control (Figure 6A, lanes 15–21). In addition, we noted that a greater amount of Mago was recovered in ΔC-PYM than in FL-PYM immunoprecipitates (Figure 6A, lanes 16 and 18), suggesting a regulatory function of the C-terminus in *Dm*PYM binding to Mago-Y14. Surprisingly, we observed only low co-precipitation of Mago with N-PYM, compared with ΔC-PYM, which contains both the N-terminal and middle domain (Figure 6A, lanes 18 and 19). This implies a role of the PYM middle domain, which itself does not bind Mago, in stabilizing the N-PYM-Y14-Mago interaction.

We next investigated the effect of the different PYM-GFP fusion proteins on EJC integrity, monitoring the ability of HA-eIF4AIII to co-IP Mago (Figure 6A, lanes 22–28). Remarkably, in spite of their differential ability to co-precipitate Mago, all three fusion proteins, FL-, N- and ΔC-PYM-GFP, displayed a similar capacity to disassemble the EJC (Figure 6A, lanes 23, 25 and 26). In contrast, neither ΔN-, M-, nor C-PYM-GFP, which failed to bind Mago, affected integrity of the EJC (Figure 6A, lanes 24, 27 and 28). These results show that the ability of the different *Dm*PYM truncations to provoke EJC disassembly correlates with their ability to bind Mago, but not with its co-precipitation efficiency.

Although both N-PYM and ΔC-PYM affected EJC stability in S2 cells (Figure 6A, lanes 25 and 26) and were equally potent in causing *oskar* mislocalization (Figure 3C, D), the two proteins differed considerably in their ability to co-precipitate Mago (Figure 6A, lanes 18 and 19). This seemingly low binding of N-PYM to Y14-Mago might reflect a short half-life of the complex. To test this hypothesis, we prepared cytoplasmic extracts from S2 cells expressing the FL-, ΔC-, N-PYM-GFP or GFP proteins, added the protein cross-linking agent DSP and performed IPs using GFP-Trap beads. The overall immunoprecipitation efficiency was reduced in the presence of DSP. However, substantially greater amounts of Mago and Y14 co-precipitated with N-PYM upon cross-linking, consistent with stabilization of the trimeric complex (Figure 6B, lane 22).

Quantification of the western blots revealed that, although the binding of both FL- and ΔC-PYM to Y14-Mago increased upon DSP treatment (1.7 and 3.6 fold, respectively), ΔC-PYM bound Y14-Mago more effectively than FL-PYM under both native and cross-linking conditions (Figure 6C). In stark contrast, the efficiency with which N-PYM co-precipitated Mago increased 92-fold (from 0.92%, to 84.88%) upon DSP cross-linking, such that it approximated that of ΔC-PYM (98.9%, Figure 6C). Hence, while the interaction of N-PYM with Y14-Mago is labile, the binding capacity of the N-terminal domain alone to the EJC is nearly equal to that of ΔC-PYM, and is far greater than that of FL-PYM. This explains the potent effect of N-PYM over-expression on EJC integrity and thus, on *oskar* RNA localization.

## Discussion

Previous studies carried out in cultured cells have led to the model that PYM - a Y14-Mago binding protein, by virtue of its association with the small ribosomal subunit, dissociates EJCs from spliced mRNAs during the first round of translation (ref. 9 and references therein). To date, however, the physiological role of PYM has remained unclear. Here we have addressed the function of PYM in an animal context.

Although a direct association of the EJC core components with *oskar* mRNA *in vivo* has been presumed [4], [14], [32], our RNA-coimmunoprecipitation experiments on *Drosophila* ovarian extracts provide the first evidence of a “physical” association of EJC core components with *oskar* mRNA. The effect of PYM on *oskar* localization can therefore be seen as a direct consequence of EJC dissociation from *oskar* RNA. This further underscores the importance of EJC association in *oskar* mRNA localization. PYM over-expression does not affect *bicoid* or *gurken* mRNA localization in the oocyte, consistent with previous genetic studies indicating no role of the EJC in this process. However, the fact that upon PYM over-expression EJCs are removed not only from *oskar*, but also from other templates such as *bicoid*, *gurken* and *nanos* mRNAs, indicates that the activity of *Dm*PYM *in vivo* is not restricted to specific mRNPs, but that the protein acts more globally on EJC-containing mRNP complexes.

Our *in vivo* analysis shows that the amount of PYM relative to the EJC core proteins, Y14 and Mago, is crucial for *Drosophila* development: PYM function is essential when the gene dosage of either of its interacting partners Y14 and Mago is reduced. Interestingly, under steady-state conditions, flies that exclusively lack *pym* function are viable and are easily maintained as a stock in the laboratory. The viability of *pym* null flies suggests either that *in vivo* the binding activity of endogenous *Dm*PYM to Y14-Mago has no physiological impact or that it may be negatively regulated. The latter hypothesis is supported by our finding that, in wild-type flies, the EJC-dependent localization of *oskar* mRNA is only abolished by expression of PYM constructs lacking the C-terminal domain. Over-expression of full-length *Dm*PYM has a mild effect that is increased in “sensitized” *osk^A87^*/+ flies, in which *oskar* RNA dosage is reduced. Such striking differences between the PYM truncations with respect to their capability to mislocalize *oskar* RNA both suggests that the full-length PYM - in contrast to its truncations - is regulated, and points to the C-terminal domain as a “key player” in such a regulatory pathway.

Studies carried out in human cell cultures showed that the C-terminal eIF2A-like domain of *Hs*PYM mediates its association with components of the 48S translation pre-initiation complex [19]. Thus an attractive hypothesis would be that, in *Drosophila*, PYM is regulated through the interaction of its C-terminal 54 residues with the small ribosomal subunit. However, none of our IP or ribosome pelleting experiments performed on *Drosophila* ovarian lysates support such an association of endogenous or ectopically expressed *Dm*PYM protein with components of the small ribosomal subunit. This is most likely due to divergence of the amino acid residues in *Dm*eIF2α (Figure S2B). Furthermore we show that over-expression of *Dm*PYM in oocytes not only impairs localization of endogenous *oskar*, but also of a non-translatable *oskar* RNA reporter. Thus a ribosomal interaction with *Dm*PYM in EJC regulation seems unlikely.

Although the interaction of *Dm*PYM with Y14-Mago is essential for EJC disassembly, the stability of the ternary complex is not important for the dissociation. The middle and the C-terminal domains of *Dm*PYM influence its interaction with Y14-Mago, albeit in opposing manners: binding is stabilized by the former and antagonized by the latter (Figure 6). Whether the middle domain somehow stabilizes the PYM N-terminus:Y14-Mago interaction or whether it promotes proper presentation of the N-terminus for Y14-Mago binding remains to be addressed. In support of an antagonistic effect of the PYM C-terminus on Y14-Mago binding, the interaction of FL-PYM with Y14 and Mago does not approximate saturation even in the presence of the protein cross-linker DSP. This clearly indicates that, *in vivo*, *Dm*PYM must be present in an equilibrium between active and dormant states. The C-terminal domain might be modified post-translationally or might serve as a binding platform for co-factors that enhance or inhibit the EJC-dismantling activity of *Dm*PYM. Further analysis, including an unbiased proteomics approach to identify novel interacting partners of *Dm*PYM, should provide insights into the regulation of *Dm*PYM and its interaction with the EJC. The fact that PYM function is essential in flies lacking one functional copy of *y14* or *mago* suggests that PYM activity is regulated by a pathway ensuring EJC homeostasis in the fly.

## Materials and Methods

### Cloning

The full length *pym* (FL-PYM) and *eIF4AIII* coding regions were PCR amplified from ovarian cDNAs using specific primers (Table S2) and cloned into pENTR/SD/D-TOPO plasmid (Invitrogen) to generate the entry clones. The PYM deletion (ΔN, ΔC, N, M and C-PYM) entry clones were generated by PCR using FL-PYM entry clone as template. The entry clones were used for recombination with the destination vector (pPFMW for N-terminal FLAG; pPWG for C-terminal eGFP; pPFMW for N-terminal FLAG-Myc) from the *Drosophila* Gateway vector collection (gift of Terence Murphy, Carnegie Institution for Science). The *eIF4AIII* entry plasmid was used for recombination with pAHW vector to generate an N-terminal HA-tagged protein. The primers are listed in Table S2.

### Fly stocks

All fly stocks were maintained at 25°C. The following fly stocks used in this study were; *w^1118^* (wild-type), *w^−^;P{lacW}wibg^SH1616^/CyO (BL#-29502)*, *w^−^;Df(2R)BSC600/SM6a* (BL#-25433), *w-,pCOGGal4::VP16;;osk^A87^*, *nosGal4::VP16/TM3Sb* [31], *w-;;nosGal4::VP16*, *w-;[bmP-BamHA]/Cyo;bgcnGFP/TM3* (gift of D. McKearin), *w^−^,GFP::Mago;;Sb/TM3Ser* [33], *y^1^scv^1^;P{TRiP.HMS01488}attP2* (BL#-35746), *y^1^scv^1^;P{TRiP.GL00515}attP40* (BL#-36096), *y^1^scv^1^;P{TRiP.GL00596}attP40* (BL#-36636). *mago^3^* [13], *tsu^Δ18^* [34] and *eIF4AIII^19^* [14] alleles were used to test genetic interaction with *pym*.

Flies lacking *pym* function were generated by crossing the recessive-lethal *pym* allele *P{lacW}wibg^SH1616^* to *w^−^; Df(2R)BSC600/SM6a*, which contains a chromosomal deletion encompassing the *bgcn* locus. The viable *pym* null adults had defective oogenesis, which was rescued by expression of a *bgcnGFP* transgene (gift of D. McKearin). The stock eventually lost the *Df(2R)BSC600* chromosome and *P{lacW}wibg^SH1616^/P{lacW}wibg^SH1616^*; *bgcnGFP* flies were used for the analyses shown in the manuscript. We refer to both genotypes (*P{lacW}wibg^SH1616^/Df(2R)BSC600;bgcnGFP* and *P{lacW}wibg^SH1616^/P{lacW}wibg^SH1616^; bgcnGFP*) as “*pym* null”, as they behaved identically in our assays (see Table S1).

For the generation of transgenic flies, pUASp-based destination plasmids containing the PYM fragments were injected together with helper plasmid as described [4].

The PYM-GFP and FLAG-GFP constructs were expressed in the germline using *nosGal4::VP16* driver. The *oskΔi(2,3)-boxB* transgene was expressed in *osk^A87^/Df(3R)^pXT103^* background.

### qRT-PCR analysis

For each biological replicate, 6 adult females (wild-type and *pym* null) were homogenized in 350 µl TRIzol LS (Invitrogen) and RNA was extracted according to the manufacturer's protocol. The RNA samples were treated with TURBO DNase I (Ambion) for 20 min and purified using RNeasy kit (Qiagen). Reverse transcription was performed using Superscript III First-Strand synthesis kit (Invitrogen) following the manufacturer's protocol. The cDNA samples were used for qRT-PCR in One-Step ABI PCR cycler using the primers listed in Table S2.

### S2 cell culture

Cells were grown in Express Five SFM Medium (Life technologies) at 25°C in the presence of penicillin-streptomycin (100 U/ml; Life technologies), puromycin (1.8 µg/ml; Sigma) and L-glutamine (2 mM; Life technologies). An S2 cell line stably transfected with pMT-Gal4-puro (gift of S. DeRenzis) was used in this study. For transient transfections, plasmids were transfected into cells in 75 cm^2^ cell culture flasks using Effectene reagent (Qiagen) following manufacturer's instructions. After 36 h, CuSO_4_ was added to 0.75 mM and the cultures incubated for 6 h for Gal4 induction. Cells were harvested using a cell scraper, washed with PBS and placed on ice for further processing.

### Co-immunoprecipitation assay and western blot analysis

All procedures were carried out at 4°C. Cytoplasmic extracts, from fly ovaries or S2 cells, were prepared using the NE-PER kit (Thermo Scientific) following manufacturer's instructions in presence of Halt protease inhibitor cocktail (Thermo Scientific). For protein cross-linking, extracts were supplemented with 1 mM DSP (Dithiobis[succinimidyl propionate], Sigma) and incubated at 4°C for 1 h with mixing. Cross-linking was stopped by addition of 50 mM Tris-HCl pH 7.8 followed by incubation for 15 min. Lysates were pre-cleared with Protein A/G beads (Roche) for 30 min. For immunoprecipitation, extracts were incubated either with rat anti-PYM (1∶250), rabbit anti-eIF4AIII (1∶150), mouse anti-FLAG (1∶100, Sigma #F3165) antibodies or with pre-blocked GFP-Trap (Chromotek) and anti-HA (Sigma) agarose beads for overnight with mixing. The beads were pre-blocked using Western Blocking reagent (Roche). For RNase treatment, 1 µl of RNase cocktail (Ambion) was added to the lysate together with the antibody. When using antibodies, the immuno-complexes were isolated by incubating the lysate with pre-blocked Protein A or G beads for 3 h. The beads were washed four times, 10 min each, with wash buffer (25 mM HEPES-KOH pH 7.5, 300 mM KCl, 4 mM MgCl_2_, 1 mM DTT, 125 mM Sucrose, 0.2% NP-40, 1× Halt protease inhibitor) and once with PBS. The beads were boiled in presence of 2× Laemmli sample buffer and the bound proteins analyzed by SDS-PAGE followed by western blotting.

The primary antibodies used for western blot staining were: Rat anti-PYM (1∶5,000; gift of E. Izaurralde), Rat anti-Y14 [11] (1∶2,500), Rabbit anti-Mago [11] (1∶2,000), Rabbit anti-eIF4AIII (1∶4,000; gift of I. Palacios), Rat anti-Btz (1∶1,000; A. Ephrussi (unpublished)), Rabbit anti-RpS6 (1∶2,000; gift of M. Hentze), Rabbit anti-RpL32 (1∶2,000; gift of M. Hentze), Rabbit anti-eIF4E (1∶2,000; gift of A. Nakamura), Rabbit anti-CBP20 (1∶2,000; gift of M. Hentze), Rabbit anti-eIF4G (1∶2,000; gift of A. Nakamura), Rabbit anti-Kinesin heavy chain (KHC, 1∶25,000; Cytoskeleton), Rabbit anti-actin (1∶2,500; Sigma), Rabbit anti-Oskar (1∶2,000), Rabbit anti-GFP (1∶2,000, Torrey Pines). Goat anti-Rabbit (1∶2,500) and anti-rat (1∶2,500) conjugated with HRP (GE Healthcare) were used as secondary antibodies.

### Quantification of co-immunoprecipitation assays

For quantification of the coIP assays using S2 cell extracts under native and DSP cross-linked conditions, signals obtained from western analysis were subjected to densitometry measurements using ImageJ (http://imagej.nih.gov/ij/) and processed with Excel (Microsoft Inc.). The relative enrichments (RE) for PYM-GFP proteins, Mago and Y14 were defined as relation of measured signals in precipitates and inputs:
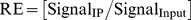
Since DSP treatment led to lower enrichments for GFP or PYM-GFP fusions (Figure 6B), all values plotted for Mago and Y14 from the individual immunoprecipitates were defined as the co-IP efficiency (CoIP_e_) relative to corresponding enrichments, estimated for GFP and GFP-PYM fusion proteins. For example, the CoIP_e_ of Mago in ΔC-PYM-GFP IP was defined as:
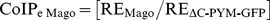



### RNA-coimunoprecipitation and RT-PCR analysis

The ovaries from fly lines co-expressing GFP-Mago and FLAG-PYM constructs were used for preparation of cytoplasmic extract using the NE-PER kit (Thermo Scientific). The extract was pre-cleared using Protein A beads for 1 h at 4°C and incubated with GFP-Trap agarose (ChromoTek) for 3 h. The beads were washed four times, 15 min each, with wash buffer containing 600 mM KCl and once with PBS. 20% of the input and beads were aliquotted for SDS-PAGE analysis and the rest processed for RNA extraction using TRI Reagent (Ambion). The recovered RNA was treated with 2 U TURBO DNase I (Ambion), and used for cDNA synthesis using SuperScript III First-Strand synthesis kit (Invitrogen) according to the manufacturer's protocol. Primers specific for *oskar*, *bicoid*, *nanos* and *gurken* were used for the PCR amplification (see Table S2). The data from 28 cycles are shown.

### Sucrose cushion centrifugation assay

Dissected ovaries were lysed in hypotonic buffer (5 mM Tris-HCl pH 7.5, 1.5 mM KCl, 2.5 mM MgCl_2_, 0.5% TX-100, 0.5% DOC, 1× Halt protease inhibitor), incubated on ice for 15 min with or without 20 mM EDTA and centrifuged at 13,000 g for 10 min. The supernatant was adjusted to the sucrose cushion buffer (10 mM Tris-HCl pH 7.5, 150 mM KCl, 5 mM MgCl_2_), layered on 1 M sucrose (200 µl extract on 700 µl sucrose solution) and centrifuged at 200,000 g for 2 h. The supernatant was concentrated using Amicon Ultra-10K filters and the pellet suspended in sucrose cushion buffer containing 1 mM DTT. The fractions were analysed on SDS-PAGE followed by western blotting.

### Whole mount immunostaining and FISH

The ovaries were dissected in PBS and processed for FISH and immunostaining as described previously [4]. For immunostaining, the primary antibodies were: Rat anti-Staufen (1∶2,500), Rabbit anti-Oskar (1∶3,000), mouse monoclonal anti-Gurken 1D12 (1∶200, Drosophila Studies Hybridoma Bank), Rat anti-PYM (1∶7,500). The DIG-labeled antisense probes to *oskar*, *bicoid* and *gurken*, used for FISH analysis has been described previously [32].

### Recombinant protein purification

The pENTRY/SD/D-TOPO vector containing FL-PYM was used for recombination with the Gateway destination vector pDEST15 (Invitrogen) to generate GST-PYM plasmid. The ΔN- and ΔC-PYM constructs were generated by PCR using primers pairs O-388/O-390 and O-389/O-391, respectively, and cloned into EcoRI and NotI site of pGEX-4T1 (gift of E. Loeser). All plasmids were fully sequenced.

The proteins were expressed in *E. coli* (BL21 DE3 Rosetta 2) and purified using glutathione beads under standard conditions, dialyzed against the following buffer: 1.5× PBS, 1 mM MgOAc, 10% Glycerol, 2 mM DTT in Spectra/Por Membrane 1 (cut-off: 6–8,000), flash-frozen in aliquots and stored at −80°C till further use.

### *In vitro* splicing assays

Preparation of DNA templates, *in vitro* transcription and preparation of embryonic nuclear extracts have been described previously [32]. Soluble nuclear extract was dialyzed against Buffer D (15 mM HEPES-KOH pH 7.9, 20% glycerol, 120 mM KCl or KOAc, 0.2 mM EDTA pH 8.0, 1 mM DTT) in Spectra/Por Membrane 1 (cut-off: 6–8,000), aliquoted, quick-frozen in liquid nitrogen and stored at −80°C.

*In vitro* splicing reactions were carried out in 25 µl, containing 10 µl of the embryo nuclear extract, ^32^P-labeled pre-mRNA substrate in a buffer (26 mM HEPES-KOH pH 7.9, 40 mM KCl, 80 mM KOAc, 4 mM MgOAc, 5 mM Creatine-Phosphate, 4 mM ATP, 2.5% PVA) for 180 min at 20°C. Purified proteins (0.5 or 1 µM of either GST, GST-FL PYM, GST-ΔN PYM or GST-ΔC PYM) were added to the 25 µl splicing reactions and further incubated for 30 min at 20°C. RNase H assays were carried out as previously described [32] using oligonucleotide Ol-25.

## Supporting Information

Figure S1 Functional characterization of endogenous PYM during *Drosophila* oogenesis and embryogenesis. (A) Western blot analysis of extracts of 0–2 hour embryos produced by wild-type (WT, lane 1) and *pym* null (lane 2) females, or females expressing shRNAs targeting *pym* (lanes 3 and 4) in the germline. Val20 and Val22 correspond to two different *pym* shRNA constructs cloned in pValium20 and pValium22 plasmids, respectively. The antibodies used for staining are indicated at the right of the panel. (B) Fluorescent immunostaining of wild-type and *pym* null ovaries during the different stages of oogenesis using anti-PYM antibody (red, left panels). DNA is stained with DAPI (cyan). Scale bar 25 µm. (C) Fluorescent *in situ* hybridization and immunostaining of stage 9 *pym* null egg-chamber using antisense riboprobes for *gurken* mRNA (red) and Gurken protein (green). *bicoid* mRNA staining (red) in a stage 11 egg-chamber is shown in (D). DAPI is in cyan. Scale bar 25 µm. (E) qRT-PCR analysis of wild-type (black boxes) and *pym* null (open boxes) adult females. The mRNAs tested are indicated on the x-axis while the y-axis shows the enrichment of mRNAs relative to 18S rRNA in arbitary units. The mRNA abundance was normalized with 18S rRNA. Data shown are from two biological replicates, each performed in triplicate. The error bars indicate standard deviation.
(TIF)

Figure S2 Domains of *Dm*PYM. (A) ClustalW alignment of the PYM amino acid sequences from *Drosophila melanogaster* (*Dm*), *Homo sapiens* (*Hs*), *Caenorhabditis elegans* (*Ce*) and *Arabidopsis thaliana* (*At*) showing amino acid residues involved in the interaction with Y14 (red asterisk) and Mago (black asterisk). The 60 amino acids at the N-terminus (aa1 - aa60) and 54 amino acids at the C-terminus (aa153 - aa207) correspond to the Y14-Mago interaction and eIF2A-like domain of *Hs*PYM, respectively. (B) Multiple sequence alignment of the C-terminus of human eIF2A (*Hs*eIF2A), human PYM. (*Hs*PYM), *Drosophila* eIF2A (*Dm*eIF2a) and *Drosophila* PYM (*Dm*PYM). The amino acid residues of human PYM showing homology with human eIF2A are marked with asterisks.
(EPS)

Figure S3 PYM transgenes and distribution of PYM-GFP fusion proteins. (A) Schematic representation of the different C-terminal GFP-tagged PYM transgenes (indicated at the right). The Y14-Mago interaction domain (black box), the eIF2A-like domain (red box) and the eGFP (green box) are indicated. The numbers represent the amino acid residues of *Dm*PYM. (B) Distribution of the GFP signal in the stage 9 egg-chambers expressing GFP-tagged PYM transgenes (indicated at the bottom of the panels). Scale bar 25 µm.
(EPS)

Figure S4 Characterization of egg-chambers over-expressing epitope-tagged *Dm*PYM transgenes. (A) Immunoblot analysis of extracts from wild-type (WT) ovaries expressing GFP-tagged PYM constructs (indicated at the top of the panel) shows a decrease of Oskar protein levels upon over-expression of ΔC-PYM and N-PYM transgenes (lanes 4 and 5, respectively). The antibodies used for staining are indicated to the right of the panel. The arrow shows endogenous PYM and an asterisk marks the position of the short isoform of Oskar protein. (B and C) Western blot analysis of ovarian extracts of *osk^A87^*/+ flies expressing FLAG-tagged and GFP-tagged FL-PYM (B) and ΔN- and ΔC-PYM (C) constructs. The FLAG-tagged ΔN- and ΔC-PYM proteins co-migrate with endogenous PYM (C, lanes 3 and 5), the latter indicated by the arrow in B and C. KHC = kinesin heavy chain. (D) Distribution of *gurken* (*grk*) and *bicoid* (*bcd*) mRNAs (shown in red) in *osk^A87^*/+ egg-chambers expressing FLAG-tagged PYM transgenes (indicated on the right), as revealed by FISH analysis. The *grk* and *bcd* transcripts were detected by antisense probe to the coding region of the respective transcripts.
(EPS)

Figure S5 Over-expressed PYM does not interact with ribosomes. Ovarian cytoplasmic extracts from *osk^A87^*/+ flies or *osk^A87^*/+ flies expressing GFP-tagged FL-, ΔN- and ΔC-PYM were analysed by immunoprecipitation using GFP-Trap beads (A) and sucrose cushion centrifugation assay (B, C). The proteins bound to the GFP beads (A, lanes 5–8) and the corresponding inputs (1%; A, lanes 1–4) were analyzed by western blotting. For sucrose cushion analysis, the extract was processed either without (B) or with the addition of 20 mM EDTA (C). The input (50%), supernatant and pellet fractions were processed for western blot analysis. The antibodies used for western blot staining are indicated at the right of the panels. The endogenous PYM is shown by the arrow. An asterisk marks a non-specific protein cross-reacting with the anti-Mago antibody. The presence of a fraction of FL-PYM protein in the ribosomal pellet could be an artefact of over-expression or represent an aggregate constituting an insoluble fraction.
(EPS)

Table S1 Summary of genetic interactions observed in *pym* loss and gain of function analysis. The columns describe the type of fly line (genetic loss or gain of function), their genotype, whether the flies were viable, fertile, and whether *oskar* mRNA was correctly localized.
(PDF)

Table S2 List of DNA primers and their sequences used in this study for cloning, RNase H protection assay, and RT-PCR and qRT-PCR analysis of RNAs.
(PDF)
