# Correction: The EJC Binding and Dissociating Activity of PYM Is Regulated in *Drosophila*

**DOI:** 10.1371/journal.pgen.1005157

**Published:** 2015-04-07

**Authors:** The PLOS Genetics Staff

Fig. 2 is mislabelled. The antibody which the authors used to detect the 40S ribosomal subunit in PYM immunoprecipitates and sucrose density fractions by western blotting (shown in Fig. 2A and 2B) is not directed against RpS6 as indicated, but against another component of the eukaryotic 40S ribosomal subunit, RACK1. The authors have provided a correct version of Fig. 2 here. In order to confirm the findings, the authors have also provided an additional S6 Fig showing a western blot of an equivalent PYM immunoprecipitation probed with RpS6 antibody.

**Fig 2:**
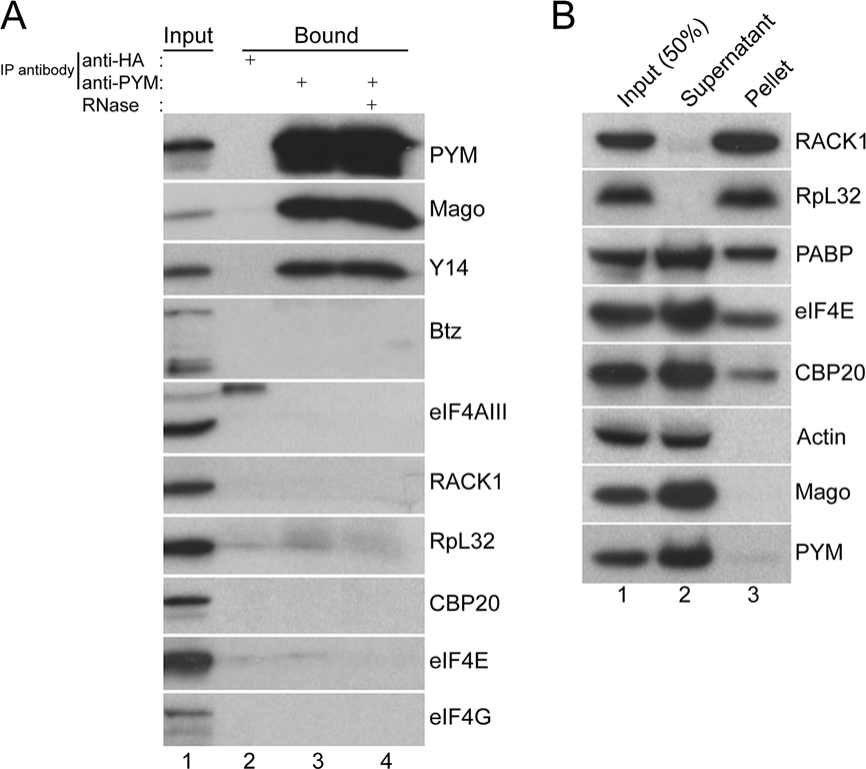
Endogenous *Dm*PYM interacts with Y14 and Mago but not with ribosomes. (A) Immunoprecipitation using anti-HA (lane 2) and anti-PYM (lane 3) antibody was performed using wild-type ovarian extracts. The precipitated proteins were analyzed by western blotting and stained with the antibodies indicated at the right of the panel. Lane 4 shows the anti-PYM precipitate from an extract treated with RNase. Input (1%) is shown in lane 1. (B) Sucrose cushion centrifugation of wild-type cytoplasmic ovarian extract. The input (lane 1; 50%), supernatant (lane 2) and pellet (lane 3) fractions were processed for western blot analysis and stained with the antibodies indicated at the right of the panel.

## Supporting Information

S6 Fig Endogenous *Dm*PYM does not interact with ribosomal proteins. Wild-type ovarian lysate was used for immunoprecipitation using either Protein A beads (lane 2) or Protein A beads coupled with anti-PYM antibody (lane 3). The precipitated proteins were stained with the antibodies as shown to the right of the panel. anti-RpS6 antibody is from Cell Signalling (mAb 54D2). Input (0.8%) is shown in lane 1.
(TIF)
