# Supplementary figures and images for: The EJC Binding and Dissociating Activity of PYM Is Regulated in Drosophila
Source: PLoS Genet. 2014 Jun 26;10(6):e1004455. doi: 10.1371/journal.pgen.1004455 (PMC4072592; doi:10.1371/journal.pgen.1004455)

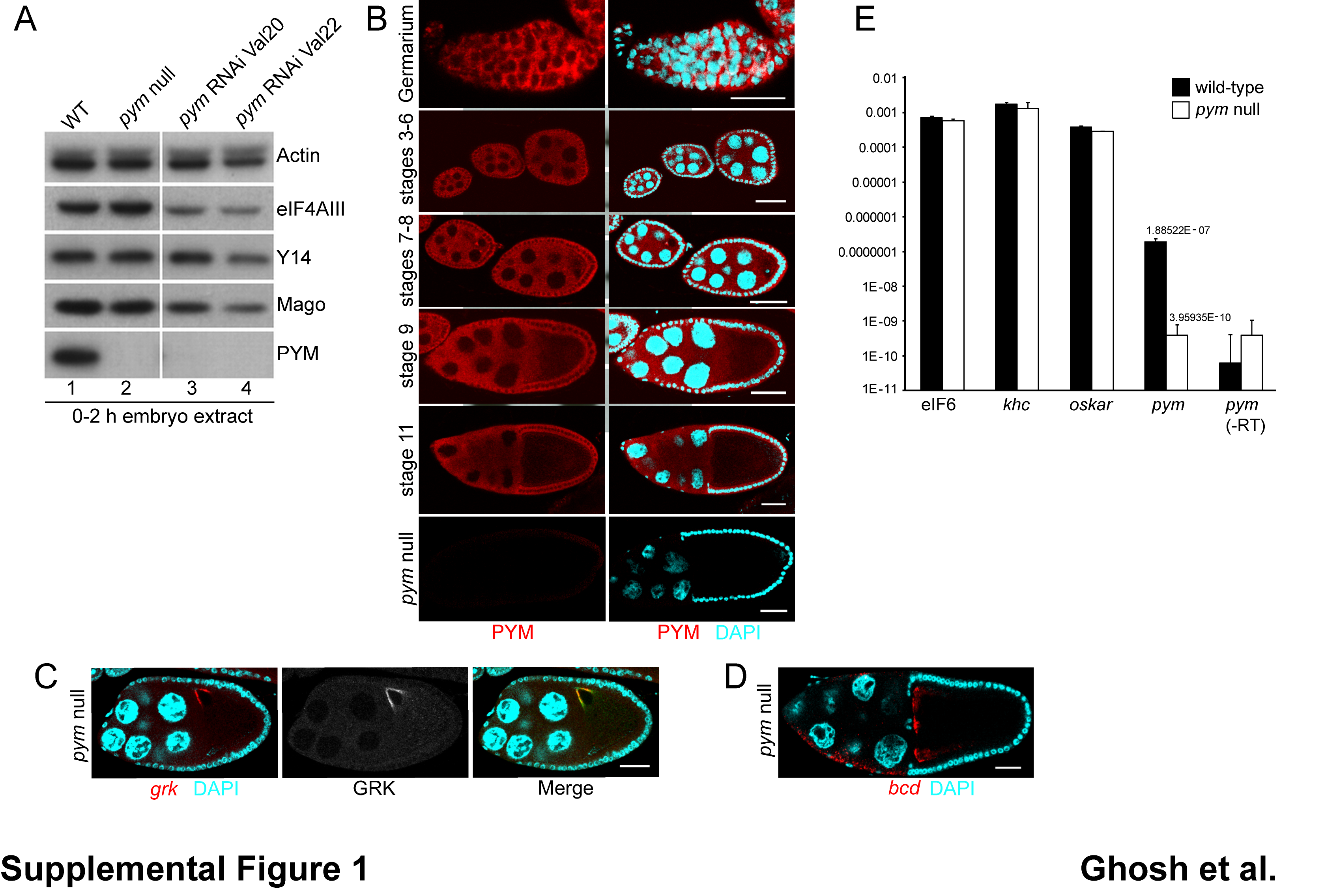

Supplement: Figure S1 — Functional characterization of endogenous PYM during Drosophila oogenesis and embryogenesis. (A) Western blot analysis of extracts of 0–2 hour embryos produced by wild-type (WT, lane 1) and pym null (lane 2) females, or females expressing shRNAs targeting pym (lanes 3 and 4) in the germline. Val20 and Val22 correspond to two different pym shRNA constructs cloned in pValium20 and pValium22 plasmids, respectively. The antibodies used for staining are indicated at the right of the panel. (B) Fluorescent immunostaining of wild-type and pym null ovaries during the different stages of oogenesis using anti-PYM antibody (red, left panels). DNA is stained with DAPI (cyan). Scale bar 25 µm. (C) Fluorescent in situ hybridization and immunostaining of stage 9 pym null egg-chamber using antisense riboprobes for gurken mRNA (red) and Gurken protein (green). bicoid mRNA staining (red) in a stage 11 egg-chamber is shown in (D). DAPI is in cyan. Scale bar 25 µm. (E) qRT-PCR analysis of wild-type (black boxes) and pym null (open boxes) adult females. The mRNAs tested are indicated on the x-axis while the y-axis shows the enrichment of mRNAs relative to 18S rRNA in arbitary units. The mRNA abundance was normalized with 18S rRNA. Data shown are from two biological replicates, each performed in triplicate. The error bars indicate standard deviation. (TIF) [file pgen.1004455.s001.tif]
