# Supplementary material for: The EJC Binding and Dissociating Activity of PYM Is Regulated in Drosophila
Source: PLoS Genet. 2014 Jun 26;10(6):e1004455. doi: 10.1371/journal.pgen.1004455 (PMC4072592; doi:10.1371/journal.pgen.1004455)
Supplement: Table S1 — Summary of genetic interactions observed in pym loss and gain of function analysis. The columns describe the type of fly line (genetic loss or gain of function), their genotype, whether the flies were viable, fertile, and whether oskar mRNA was correctly localized. (PDF) [file pgen.1004455.s006.pdf]

Table S1: Summary of genetic interactions observed in *pym* loss and gain of function analysis

Fly Line	Genotype	Viability	Female Fertility	*oskar* Localization
pym null	*w-; P{lacW}wibg*^{SH1616}*; bgcnGFP*<br>*w-;P{lacW}wibg*^{SH1616}*/Df(2R)BSC600;bgcn*GFP	yes	yes	yes
pym null, *y14* heterozygous	*w-; P{lacW}wibg*^{SH1616}*, tsu*$^{\Delta 18}$*/Df(2R)BSC600; bgcnGFP*	no	n.a.	n.a.
pym null, *mago* heterozygous	*w-; P{lacW}wibg*^{SH1616}*, mago*^{3}*/Df(2R)BSC600; bgcnGFP*	no	n.a.	n.a.
pym null, *eIF4AIII* heterozygous	*w-; P{lacW}wibg*^{SH1616}*/Df(2R)BSC600; eIF4AIII*^{19}*, bgcnGFP*	yes	yes	n.d.
FL-PYM over-expression in WT egg chambers	*w-; P{UAS-FL-PYM-GFP}; nosGal4::VP16*	yes	yes	no; partial mislocalization
ΔN-PYM over-expression in WT egg chambers	*w-; P{UAS-ΔN-PYM-GFP}; nosGal4::VP16*	yes	yes	yes
ΔC-PYM over-expression in WT egg chambers	*w-; P{UAS-ΔC-PYM-GFP}; nosGal4::VP16*	yes	no	no
N-PYM over-expression in WT egg chambers	*w-; P{UAS-N-PYM-GFP}; nosGal4::VP16*	yes	no	no
M-PYM over-expression in WT egg chambers	*w-; P{UAS-M -PYM-GFP}; nosGal4::VP16*	yes	yes	yes
C-PYM over-expression in WT egg chambers	*w-; P{UAS-C-PYM-GFP}; nosGal4::VP16*	yes	yes	yes
FL-PYM over-expression in *oskar* heterozygotes	*w-, pCOGGal4::VP16; P{UAS-FL -PYM-GFP}; osk*^{A87}*, nosGal4::VP16*	yes	no	no
ΔN-PYM over-expression in *oskar* heterozygotes	*w-, pCOGGal4::VP16; P{UAS-ΔN-PYM-GFP}; osk*^{A87}*, nosGal4::VP16*	yes	yes	yes

ΔC-PYM over-expression in *oskar* heterozygotes	*w-, pCOGGal4::VP16; P{UAS-ΔC-PYM-GFP}; osk^{A87}, nosGal4::VP16*	yes	no	no

WT = wild-type, n.a = not applicable, n.d. = not determined
