# Supplementary material for: The EJC Binding and Dissociating Activity of PYM Is Regulated in Drosophila
Source: PLoS Genet. 2014 Jun 26;10(6):e1004455. doi: 10.1371/journal.pgen.1004455 (PMC4072592; doi:10.1371/journal.pgen.1004455)
Supplement: Table S2 — List of DNA primers and their sequences used in this study for cloning, RNase H protection assay, and RT-PCR and qRT-PCR analysis of RNAs. (PDF) [file pgen.1004455.s007.pdf]

Table S2: List of primers

Primer Name	Sequence (from 5' to 3')	Used for
FL-Fwd	CACCATGAGCACGTACCTACAGAGCAG	FL- and N-PYM cloning in pENTR/SD/D-TOPO vector
FL-Rvs	TGAGCGCGGCGTGCTC	FL- and C-PYM cloning in pENTR/SD/D-TOPO vector
DN-Fwd	CACCATGTGTCCCCTGCTGGC	DN- and M-PYM cloning in pENTR/SD/D-TOPO vector
DC-Rvs	GGCAGGGTCCACCACCTC	DC- and M-PYM cloning in pENTR/SD/D-TOPO vector
N-Rvs	GCCAGGCGGCACTCC	N-PYM cloning in pENTR/SD/D-TOPO vector
C-Fwd	CACCATGAAGCAGTTGAAGAAGCTGCG	C-PYM cloning in pENTR/SD/D-TOPO vector
eIF4AIII-Fwd	CACCATGGCGCGCAAGAATGC	eIF4AIII cloning in pENTR/SD/D-TOPO vector
eIF4AIII-Rvs	GATCAAGTCAGCCACGTTCATGG	eIF4AIII cloning in pENTR/SD/D-TOPO vector
oskar-Fwd	GCAACTATATATCCGTGCGCG	RT-PCR
oskar-Rvs	CCCGTCAGTTTTCGATATTCA	RT-PCR
bicoid-Fwd	GCTCTTGTCCAGACCCTTCAAAGG	RT-PCR
bicoid-Rvs	TGGGTCGACCAATGTCAATGGCG	RT-PCR
gurken-Fwd	CGCCTACAAGAGCTGGAATG	RT-PCR
gurken-Rvs	ACATTGCGGAGCATGAAAAC	RT-PCR
nanos-Fwd	CAGCAAACGGACGAGATTG	RT-PCR
nanos-Rvs	ACATGCGACCGAGATCATC	RT-PCR
eGFP-Fwd	CACCATGGTGAGCAAGGGC	eGFP cloning in pENTR/SD/D-TOPO vector
eGFP-Rvs	CTTGTACAGCTCGTCCATGC	eGFP cloning in pENTR/SD/D-TOPO vector
OL-25	TGATTCCATTCT	RNase H protection assay
O-388-Fwd	CCCGAATTCTGTCCCCTGCTGGCCGCCGAG	DN-PYM cloning in pGEX-4T1, EcoRI site is in bold
O-389-Fwd	CCCGAATTCAGCACGTACCTACAGAGCAGCGAG	DC-PYM cloning in pGEX-4T1, EcoRI site is in bold
O-390-Rvs	CCCGCGGCCGCCTATGAGCGCGGCGTGCTCTC	DN-PYM cloning in pGEX-4T1, NotI site is in bold
O-391-Rvs	CCCGCGGCCGCCTAGGCAGGGTCCACCACCTCCTGGG	DC-PYM cloning in pGEX-4T1, NotI site is in bold
eIF6-Fwd	AGGCTCAGTCAGGCCAGTAA	qRT-PCR
eIF6-Rvs	TCGTCGTTGTTCTCGAATTG	qRT-PCR
khc-Fwd	AAAGTGGTCTGCCGATTCC	qRT-PCR
khc-Rvs	AATACACCTTGCCCGCTATG	qRT-PCR

oskar-Fwd	AAGCGAATGCTGTCACCTAAA	qRT-PCR
oskar-Rvs	GGTGAGGCCTGAAAGCAAT	qRT-PCR
pym Fwd	GCGATTCCCCATTCGTATAA	qRT-PCR
pym Rvs	CCTCTGCACGAAAGAACACA	qRT-PCR
18s Fwd	CGGAGAGGGAGCCTGAGAA	qRT-PCR
18s Rvs	AGCTGGGAGTGGGTAATTTACG	qRT-PCR
