# Supplementary figures and images for: Correction: The EJC Binding and Dissociating Activity of PYM Is Regulated in Drosophila
Source: PLoS Genet. 2015 Apr 7;11(4):e1005157. doi: 10.1371/journal.pgen.1005157 (PMC4388473; doi:10.1371/journal.pgen.1005157)

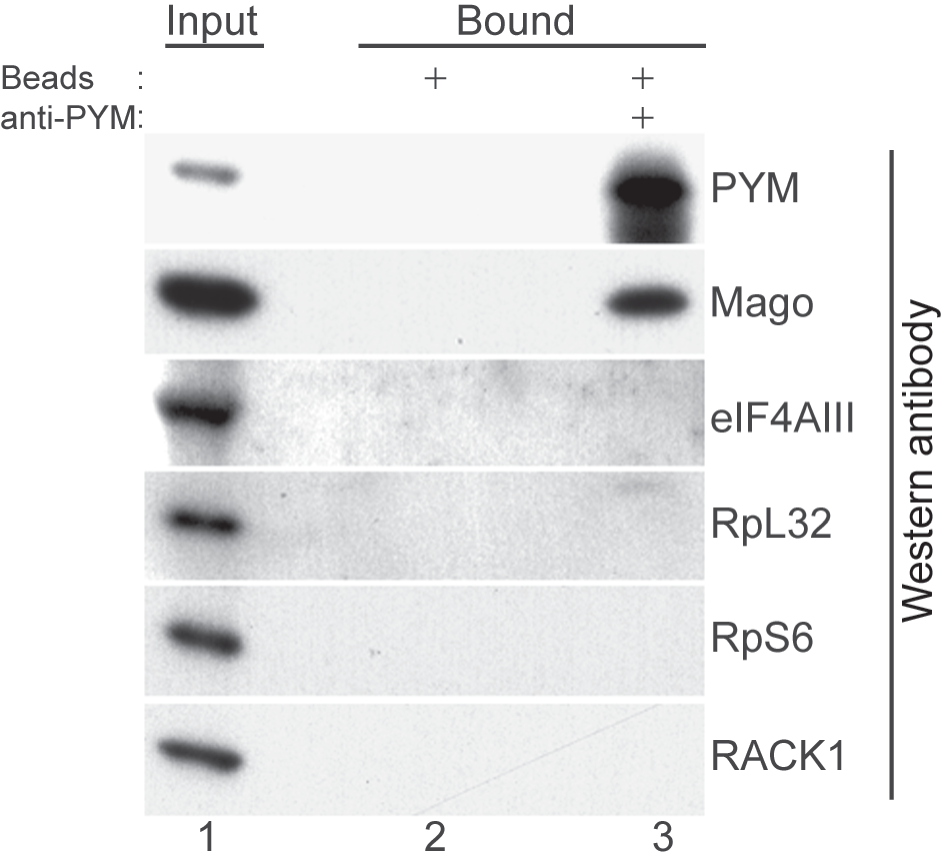

Supplement: S6 Fig — Wild-type ovarian lysate was used for immunoprecipitation using either Protein A beads (lane 2) or Protein A beads coupled with anti-PYM antibody (lane 3). The precipitated proteins were stained with the antibodies as shown to the right of the panel. anti-RpS6 antibody is from Cell Signalling (mAb 54D2). Input (0.8%) is shown in lane 1. (TIF) [file pgen.1005157.s001.tif]
